# Alteration of nanomechanical properties of pancreatic cancer cells through anticancer drug treatment revealed by atomic force microscopy

**DOI:** 10.3762/bjnano.12.101

**Published:** 2021-12-14

**Authors:** Xiaoteng Liang, Shuai Liu, Xiuchao Wang, Dan Xia, Qiang Li

**Affiliations:** 1 School of Materials Science and Engineering, Hebei University of Technology and Tianjin Key Laboratory of Materials Laminating Fabrication and Interface Control Technology, Tianjin, 300130, China; 2 Key Laboratory of Colloid and Interface Chemistry of Ministry of Education, And School of Chemistry and Chemical Engineering, Shandong University, Jinan, 250100, Shandong, China; 3 Tianjin Medical University Cancer Institute & Hospital, Tianjin, 300060, China

**Keywords:** anticancer drug, atomic force microscopy, nanomechanical properties, pancreatic cancer cells

## Abstract

The mechanical properties of cells are key to the regulation of cell activity, and hence to the health level of organisms. Here, the morphology and mechanical properties of normal pancreatic cells (HDPE6-C7) and pancreatic cancer cells (AsPC-1, MIA PaCa-2, BxPC-3) were studied by atomic force microscopy. In addition, the mechanical properties of MIA PaCa-2 after treatment with different concentrations of doxorubicin hydrochloride (DOX) were also investigated. The results show the Young's modulus of normal cells is greater than that of three kinds of cancer cells. The Young's modulus of more aggressive cancer cell AsPC-1 is smaller than that of less aggressive cancer cell BxPC-3. In addition, the Young's modulus of MIA PaCa-2 rises with the increasing of DOX concentration. This study may provide a new strategy of detecting cancer, and evaluate the possible interaction of drugs on cells.

## Introduction

Pancreatic cancer is a highly malignant tumor [1] with the highest mortality rate (ca. 100%) and the lowest 5-year survival rate (≤5%) when all stages are combined [2–3]. Since no significant symptoms appear until the late stage of pancreatic cancer, the development of early diagnosis methods is of importance. The traditional biological methods to research pancreatic cancer are based on molecular genetics and gene signaling. However, the mechanical properties of cells, which can enable cells to express various biological functions have been ignored [4]. Recently, changes of the physical properties of cells are considered to be signs of malignant transformation to cancer cells [5–6]. Measuring the difference in mechanical properties between cancer and normal cells is of great help to understand the changes related to cancer, and may provide the possibility for the early diagnosis of cancer [7].

In recent decades, anticancer drugs have been developed in great number, enabling the control and treatment of many cancers to improve life quality and life span of people. Many approved anticancer drugs have significant effects on cell membrane proteins and/or the cytoskeleton, which cause cancer cell death [8–9]. Fang et al. found that *N*-methyl-ᴅ-aspartate (NMDA) binding to NMDA receptors on cell membranes will increase the overall contractile forces in the cytoskeleton, thus increasing the pre-existing mechanical stress [10]. Yun et al. reported that the physical properties of HeLa cells treated by docetaxel are different from that of untreated ones [9]. Therefore, the study of drug–cell interaction regarding cellular mechanics could be an effective way for drug evaluation. Important information, including drug efficacy and safety can be obtained from measuring the alteration of cellular mechanics, which provides a guide for the innovation and development of anticancer drugs [11].

Atomic force microscopy (AFM) has matured into a forceful nanoscale platform for imaging biological samples and quantifying biomechanical properties of living cells under (almost) physiological conditions in situ. It offers nanoscale force sensitivity, the ability to work in liquid phases, and requires no staining [12–14]. With the development of AFM, researchers have been able to conduct extensive research on biological issues through imaging the ultrastructure of living cells [15–16], cell membranes, membrane proteins [17–18] and DNA [19], and through recording single molecular force spectra [20–21]. However, the morphology and the nanoscale mechanical properties of malignant pancreatic cancer cells (PCCs) under anticancer drug treatment have not been elucidated. Such elucidation could hint to possible early ways of diagnosis and efficient drugs for controlling or even curing pancreatic cancer.

Herein, nanostructure and Young's modulus of normal and PCCs were measured with AFM. The results illustrate that the Young's modulus of normal cells (HDPE6-C7) is greater than that of three lines of cancer cells (AsPC-1, MIA PaCa-2, and BxPC-3). In addition, the mechanical properties of MIA PaCa-2 cells treated with different concentrations of doxorubicin hydrochloride (DOX) were also investigated. An increased Young's modulus after treatment with increasing DOX concentrations was shown. This study may be conducive regarding innovations in cancer prevention, diagnosis methods, and the application of drug screening.

## Materials and Methods

### Materials

Dulbecco’s modified eagle medium (DMEM), RPMI-1640 and phosphate-buffered saline (PBS) were obtained from Shijiazhuang Hongwei Biotechnology Co., Ltd. AF488-WGA and Hoechst 33342 were purchased from Thermo Fisher Scientific. All medicals and reagents were used without further treatment.

### Cell culture

HPDE6-C7, AsPc-1 and BxPC-3 cells were cultured in RPMI-1640 with 10% fetal bovine serum and 1% double antibody. MIA PaCa-2 was cultured in DMEM high-glucose medium with 10% fetal bovine serum, 2.5% horse serum, 1% sodium pyruvate and 1% double antibody. Cells are cultured in a humidified atmosphere at 37 °C with 5% CO_2_. For the cell–drug interaction, DOX in different concentrations (10, 30, and 50 µg/mL) was added into MIA PaCa-2, which was pre-grown for two days. The DOX solution was removed after 4 h and the cells were then washed with PBS for three times. Afterwards, the cells were cultured in normal medium for another 12 h.

### Laser confocal microscopy

The cells (3 × 10^4^ cells/well) were grown in 500 µL culture medium for 24 h in a four-chambered confocal culture dish. The original medium was removed after the cells were fixed to the dish. Then, the cells were washed with PBS for three times. Afterwards, the cells were fixed with 4% paraformaldehyde for 15 min and washed with PBS (25 °C). Cell nuclei and membranes were stained with Hoechst 33342 (1 µg/mL) or AF488-WGA (1 µg/mL) for 10 min. Then, the cells were rinsed three times with PBS before imaging with laser confocal microscopy. To investigate the effect of DOX on the nanostructure of MIA-PaCa-2, cell nuclei and the cytoskeleton were stained with DAPI (10 µg/mL) and FITC-Phalloidin (100 nM), respectively, for 30 min and 5 min. The cytoskeleton and cell nuclei were investigated by laser confocal microscopy.

### AFM measurement

**Figure 1:**
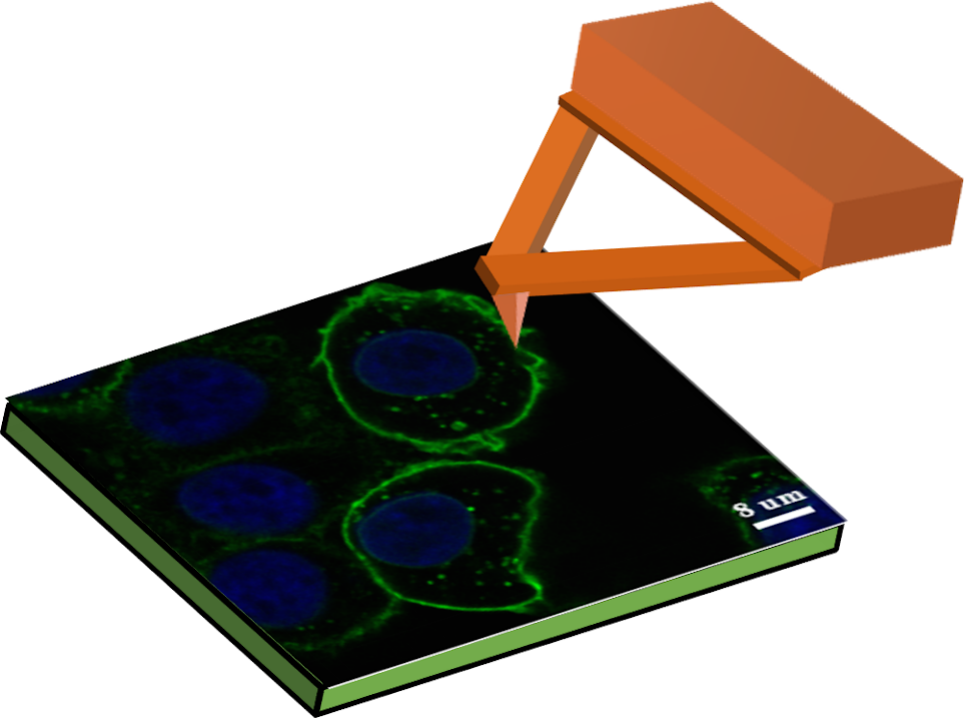
Schematic image of the pancreatic cell characterized by AFM.

The slides with grown cells were transferred to the sample table. Then, culture medium was added both to the cell slide surface (50 µL) and the AFM tip (10 µL). The characterization was carried out using a Cypher ES AFM (Asylum Research, USA) at room temperature with soft cantilevers (TR400PSA-L) with a resonance frequency of approx. 11 kHz and a spring constant of approx. 0.02 N/m. The schematic diagram of the cells characterized by AFM is shown in Figure 1. For the mechanical mapping, the AFM cantilever needs to be calibrated first. During the scanning process, the applied force should be less than 3 nN to prevent cell destruction. For force mapping, 400 force curves were collected for each selected area and at least 30 cells of each type were measured for statistical analysis.

The Hertz model is used in the calculation of cell mechanical properties. The force (*F*) exerted by the probe on the cell can be expressed by the following equation,


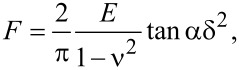


where *E* is the Young’s modulus, ν is the poisson ratio, α is the half-opening angle of the probe, and δ is the indentation depth. Thus the *E* can be calculated by transforming the above equation:


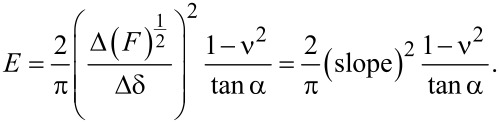


Hence the Young’s modulus can be calculated by fitting the linear part of the force–distance curves, that is, the slope of the force–distance curve.

Energy dissipation is the loss of mechanical energy during each trace–retrace cycle. The hysteresis in the force–distance curves between different types of cells indicates the energy dissipation. The dissipated energy can be calculated by the following formula,


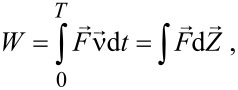


where *W* is the total amount of energy dissipation, and its value in the force–distance curve is the difference between the area of the retrace and trace curves.

### Statistical analysis

The Young's modulus of MIA PaCa-2 after adding different concentrations of DOX was presented as mean ± standard deviation and tests were carried out for the statistical analysis. (***) represents *p* < 0.001.

## Results and Discussion

### The nanostructure of different types of cells

**Figure 2:**
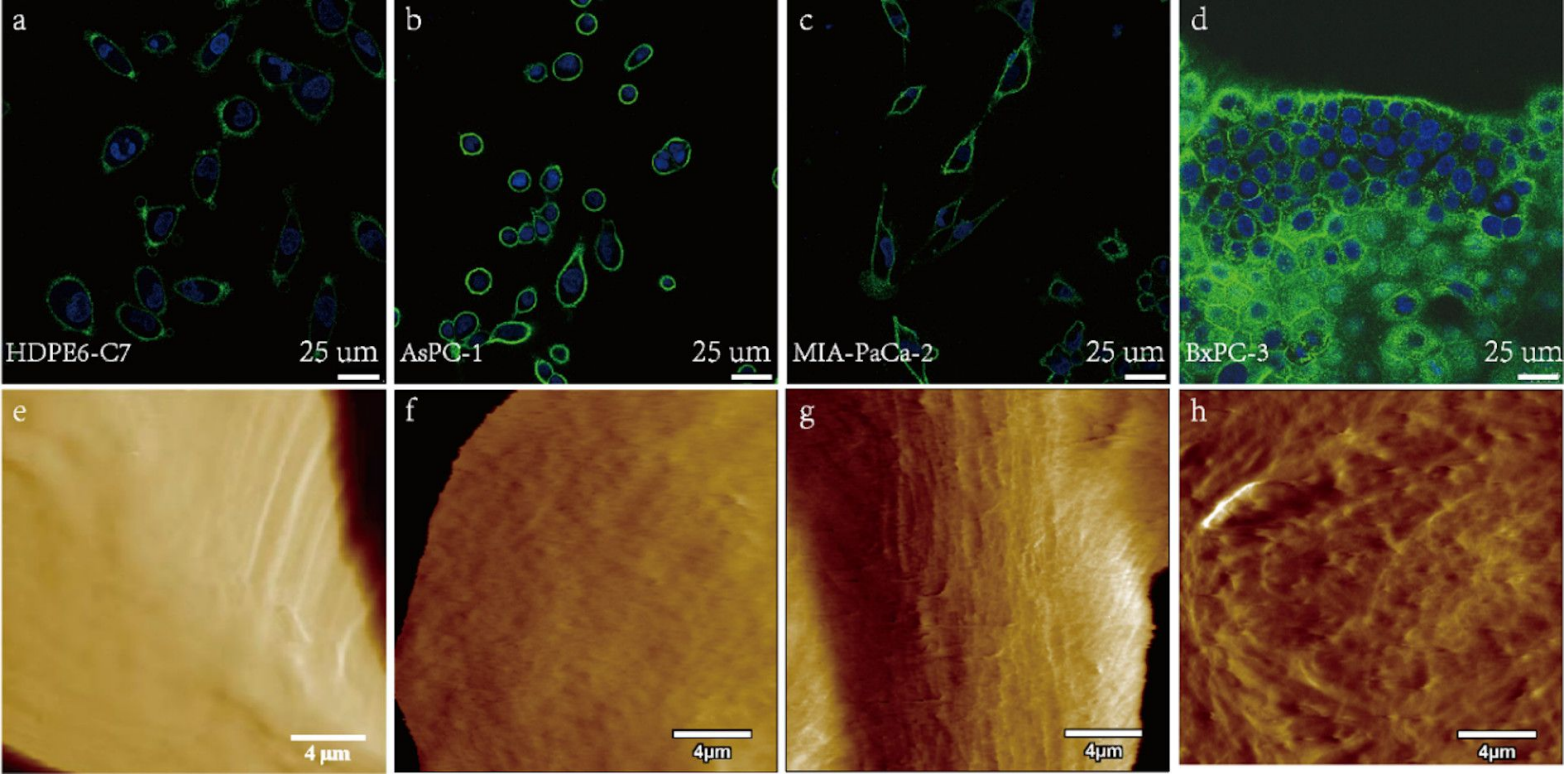
Laser confocal fluorescence images (a–d) and deflection maps (e–h) of (a, e) HDPE6-C7; (b, f) AsPC-1; (c, g) MIA-PaCa-2, and (d, h) BxPC-3.

The morphology of different types of cells characterized by laser confocal microscopy is shown in Figure 2a–d. It is apparent that HPDE6-C7 cells are oval or round with a cell size of tens of micrometers (Figure 2a). BxPC-3 cells aggregate and grow in a round shape (Figure 2b), which is quite different from HDPE6-C7 cells. However, the morphology of AsPC-1 cells (Figure 2c) and MIA-PaCa-2 cells (Figure 2d) is not significantly different from that of HDPE6-C7. Therefore, it is not feasible to identify cancer cells only by cell morphology. Moreover, in actual cases, it may happen that cancer cells mimic the morphology of normal cells [22–24].

The nanostructure of the different types of cells measured by AFM is shown in Figure 2e–h. The nanostructures of the four cell types are significantly different. The detailed fibrous microstructure can be seen on the surface of BxPC-3 cells (Figure 2h) due to the smooth cell surface. However, the detailed surface structures of the remaining three types of cells is hardly seen (Figure 2e–g) because of the enormous height difference between the substrate and the cell surface. The sharp contrast reduced the details of the cell surface microstructure of HDPE6-C7, AsPC-1, and MIA-PaCa-2 cell lines. The corresponding analyses of cell surface roughness is listed in Supporting Information File 1, Table S1, which shows that the surface roughness of MIA-PaCa-2, HDPE6-C7, and AsPC-1 is larger than that of BxPC-3, consistent with the result of surface tomography. Although a nanostructure difference exists among these four types of cells, it is also difficult to distinguish cancer cells from normal ones.

### The fingerprint nanomechanical properties of various cells

**Figure 3:**
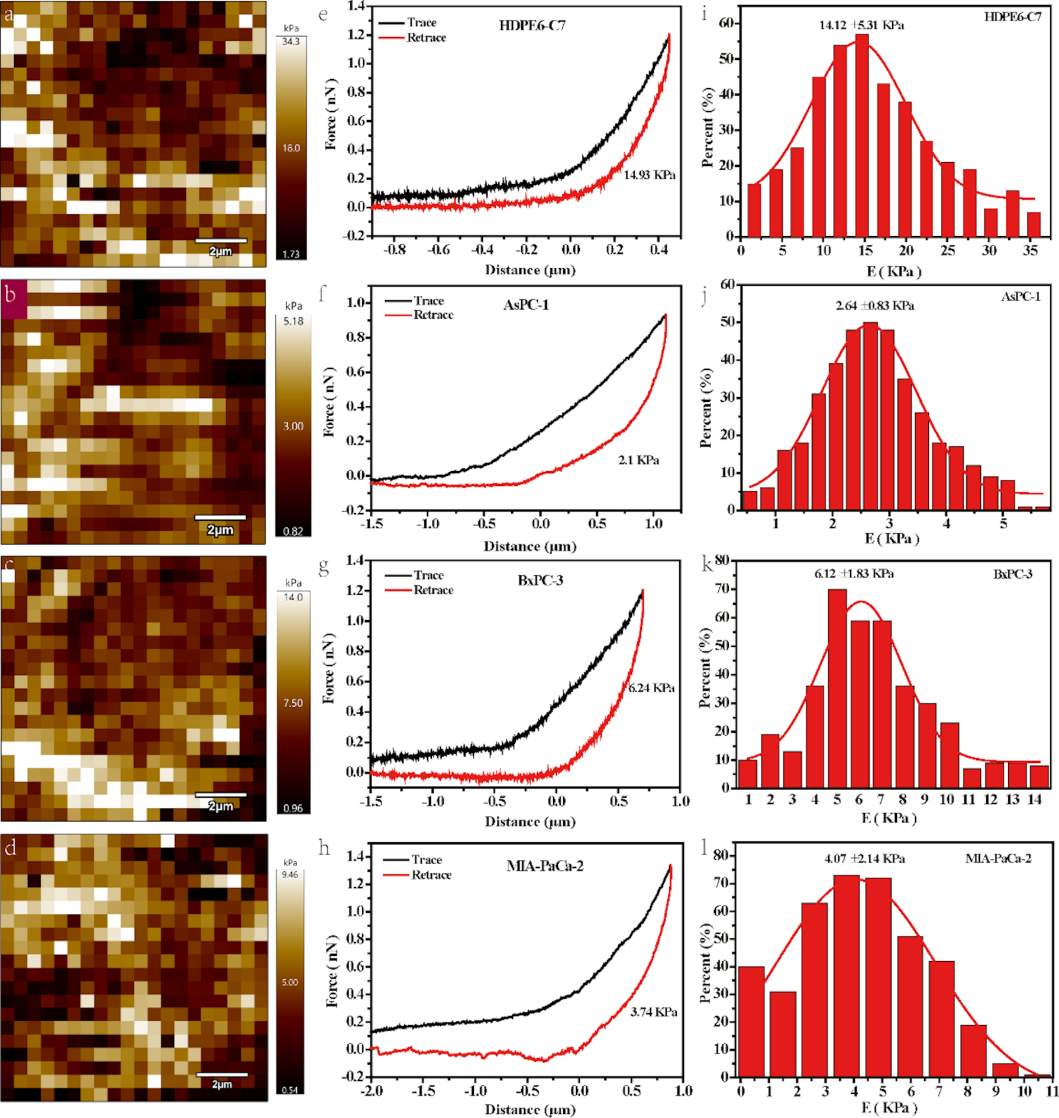
Nanomechanical mapping, FDCs, and corresponding Young's modulus distribution of (a) HDPE6-C7, (b) AsPC-1, (c) BxPC-3, and (d) MIA-PaCa-2.

To distinguish the cancer cells from the normal ones, the mechanical properties underneath the topography of different cells were evaluated. Figure 3 shows the nanomechanical mapping, typical force–distance curve and the corresponding Young’s modulus distributions of single cells of different types. For the nanomechanical mapping, brighter colors mean a higher Young’s modulus while darker colors mean a lower Young’s modulus (Figure 3a–d). The Young’s modulus of cell surfaces is not homogenously distributed. Figure 3e–h show the force–distance curves (FDCs) of different cells. It demonstrates that the HPDE6-C7 forms a relatively linear FDC while the other three cancer cell lines form nonlinear FDCs, which can be evaluated regarding the slope of the FDCs [25]. The Young’s modulus of the four kinds of cells obtained by fitting the linear part of the withdrawal curves are 14.93, 2.1, 6.24 and 3.74 kPa, which indicates that pancreatic cancer softens pancreatic cells. Also, there is energy dissipation manifested as hysteresis in a cycle of force–distance curves in all four kinds of cells. The results (Supporting Information File 1, Table S2) show that the hysteresis in the force–displacement cycle of HPDE6-C7 is smaller than that of the three PCCs. This could be caused by the difference of the internal friction and/or vicious damping [26–27] between the normal and the cancer cells. The relative Young’s modulus distributions of different kinds of cells, according to the nanomechanical mapping (Figure 3a–d) and the FDCs (Figure 3e–h) are given in Figure 3i–l. The Young's modulus of HPDE6-C7 (14.12 ± 5.31 kPa) is larger than that of AsPC-1 (2.64 ± 0.83 kPa), BxPC-3 (6.12 ± 1.83 kPa), and MIA-PaCa-2 (4.07 ± 2.14 kPa), which is consistent with the results obtained from the single force–distance curves.

Due to measurement errors, it is not accurate to utilize the Young's modulus of one single cell to represent the nanomechanical properties of one cell type. Therefore, the Young’s modulus of different cells (≥30) of the same cell type were measured to obtain more accurate cell mechanical properties. Supporting Information File 1, Figure S1 shows the point distribution of Young’s modulus of different kinds of cells. It illustrates that the point distributions of the Young’s modulus of the three types of PCCs (Figure S1a–c) are more compact than that of the normal pancreatic cells HDPE6-C7 (Figure S1d). It may be caused by the more complex distribution of biomolecules, such as proteins and sugars, existing on the normal cell membrane surface than that on the cancer cell surface [28–30].

**Figure 4:**
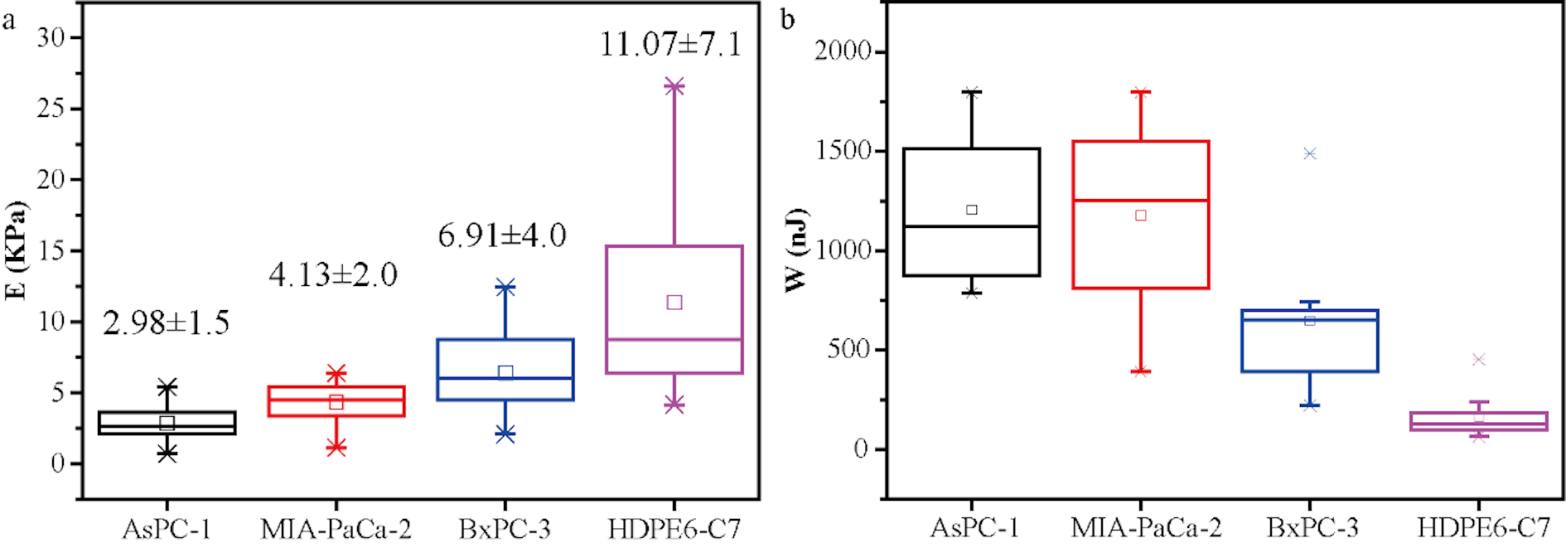
Statistics of (a) Young's modulus and (b) energy dissipation for AsPC-1, MIA-PaCa-2, BxPC-3, and HDPE6-C7.

The statistics of the Young's modulus values of the four kinds of cells are illustrated in Figure 4a. The Young’s modulus of normal pancreatic cells HDPE6-C7 is the highest (11.07 ± 7.1 kPa), compared to the three kinds of PCCs, BxPC-3 cells (6.91 ± 4 kPa), MIA PaCa-2 cells (4.13 ± 2 kPa), and AsPC-1 cells (2.98 ± 1.5 kPa). In addition, the nanomechanical properties are also different among the three kinds of cancer cells. The Young’s modulus of AsPC-1 cells is the smallest while that of the BxPC-3 cells is the highest. This is because AsPC-1 cells are metastatic cancer cells, which are more aggressive than the in situ cancer cells BxPC-3. This finding is consistent with previously reported results [12,31–32]. The statistical comparison of the energy dissipation in the four types of cell lines is shown in Figure 4b. The energy dissipation in the normal cell line (HDPE6-C7) is smaller than that in the PCCs (AsPC-1, MIA PaCa-2, and BxPC-3) and the stiffer the cells, the larger the energy dissipation. This indicates that the energy dissipation may become another indicator for the different nanoscale mechanical responses of the cells. Therefore, the nanomechanical property could be a fingerprint to distinguish PCCs from normal pancreatic cells. AFM-based techniques could be developed for the diagnosis of pancreatic cancer based on the differences in Young's modulus in the early stage.

### The effect of anticancer drugs on the nanomechanical property of PCCs

**Figure 5:**
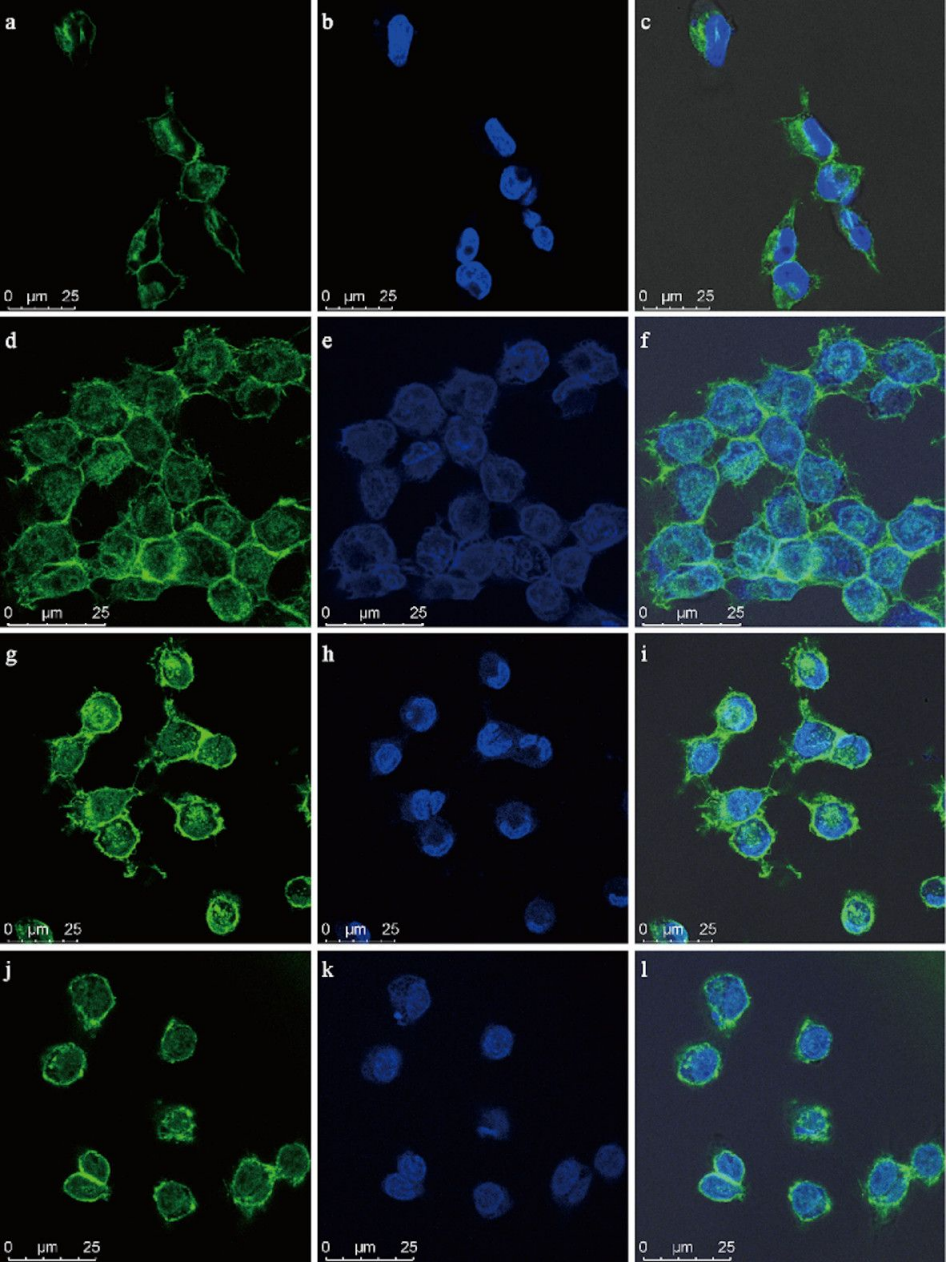
Laser confocal fluorescence images of MIA PaCa-2 before and after DOX treatment. (a–c) Without DOX treatment; (d–f) treated with 10 µg/mL DOX; (g–i) treated with 30 µg/mL DOX; (j–l) treated with 50 µg/mL DOX. Cells were stained for nuclei (blue) and cytoskeleton (green).

**Figure 6:**
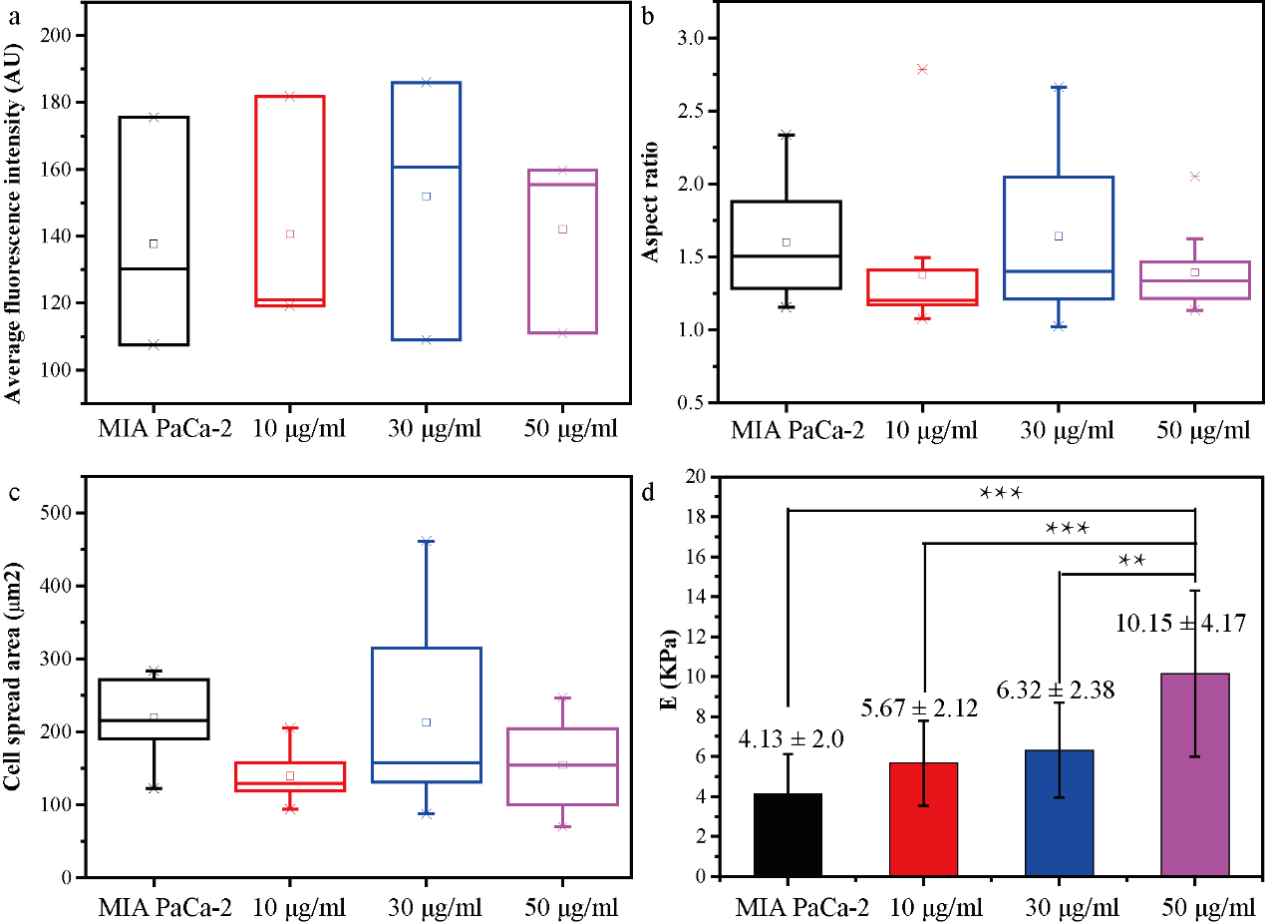
The statistic comparison of the nanostructure and the nanomechanical properties of MIA PaCa-2 cells treated with different concentrations of DOX. (a) The average fluorescence intensity of the cytoskeleton, (b) aspect ratio, (c) cell spread areas and (d) the corresponding Young's modulus of MIA PaCa-2 cells with/without DOX treatment.

The effect of anticancer drugs on the cellular mechanics provides a new way for drug evaluation and even provides credible guidance for the innovation and development of anticancer drugs. The laser confocal fluorescence images of MIA PaCa-2 before and after treating with DOX in different concentrations are shown in Figure 5. The results show that the cell morphologies are not affected significantly by DOX. The quantitative analysis of the average fluorescence intensity of the cytoskeleton, aspect ratio, and cell spread areas of MIA PaCa-2 with/without DOX treatment are compared in Figure 6a–c. All indicators show no significant morphology variations before and after DOX treatment. The corresponding nanomechanical properties of MIA PaCa-2 treated with DOX in different concentrations are shown in Supporting Information File 1, Figure S2. It demonstrates that the point distribution of Young's modulus of untreated MIA PaCa-2 is compact (Figure S2a). After adding DOX, the variation range of the Young's modulus of MIA PaCa-2 cells became wider with the DOX concentration increasing from 10 to 50 µg/mL (Figure S2b–d). Concurrently, the Young's modulus value rises with increasing DOX concentration. It may be because DOX increases the contractile forces among the cytoskeleton and the amount of microtubules [9–10].

The statistics of the Young's modulus values of MIA PaCa-2 treated with different concentrations of DOX were obtained (Figure 6d). The Young's modulus of MIA PaCa-2 cells increases with increasing concentration of DOX. The Young's modulus of untreated MIA PaCa-2 cells was 4.13 ± 2.0 kPa, while that of treated MIA PaCa-2 cells with 10, 30, and 50 µg/mL DOX was 5.67 ± 2.12 kPa, 6.32 ± 2.38 kPa, and 10.15 ± 4.17 kPa, respectively, an increase by 37.29%, 53.03%, and 145.76%, respectively, compared with the uncreated samples. It is obvious that the nanomechanical properties of MIA PaCa-2 cells are significantly changed by adding DOX in different concentrations, which is believed to enable an estimation of the internal structural changes of MIA PaCa-2 cells treated with DOX. This technique could be utilized to evaluate the anticancer drug effect on PCCs in patients treated with anticancer drugs even when the cell morphology does not change significantly.

## Conclusion

In conclusion, the nanostructure and physical properties of normal cells and PCCs were revealed by AFM. The Young's modulus of normal cells is larger than that of three kinds of PCCs. Moreover, the Young's modulus of the more aggressive cancer cells (AsPC-1) is smaller than that of the less aggressive ones (BxPC-3). In addition, the nanomechanical properties of MIA PaCa-2 cells treated with an anticancer drug were also studied. The Young's modulus of MIA PaCa-2 cells showed an increasing trend with increasing concentration of DOX. Since nanomechanical properties can be used as an indicator to identify cancer cells from normal ones, this research may provide a new method for early screening of cancer. Also, the nanomechanical property variation after treatment with the anticancer drug could be helpful for improving the efficiency of drug screening and development.

## Supporting Information

File 1 Surface roughness, energy dissipation, point distribution of Young's modulus of AsPC-1, MIA-PaCa-2, BxPC-3, and HDPE6-C7 and point distribution of MIA PaCa-2’s Young's modulus treated by DOX in different concentrations.
