# Supplementary material for: Alteration of nanomechanical properties of pancreatic cancer cells through anticancer drug treatment revealed by atomic force microscopy
Source: Beilstein J Nanotechnol. 2021 Dec 14;12:1372–9. doi: 10.3762/bjnano.12.101 (PMC8685561; doi:10.3762/bjnano.12.101)
Supplement: File 1 — Surface roughness, energy dissipation, point distribution of Young's modulus of AsPC-1, MIA-PaCa-2, BxPC-3, and HDPE6-C7 and point distribution of MIA PaCa-2’s Young's modulus treated by DOX in different concentrations. [file Beilstein_J_Nanotechnol-12-1372-s001.pdf]

BEILSTEIN JOURNAL OF NANOTECHNOLOGY

Supporting Information

for

Alteration of nanomechanical properties of pancreatic cancer cells through anticancer drug treatment revealed by atomic force microscopy

Xiaoteng Liang, Shuai Liu, Xiuchao Wang, Dan Xia and Qiang Li

Beilstein J. Nanotechnol. **2021,** *12,* 1372–1379. doi:10.3762/bjnano.12.101

Surface roughness, energy dissipation, point distribution of Young's modulus of AsPC-1, MIA-PaCa-2, BxPC-3, and HDPE6-C7 and point distribution of MIA PaCa-2’s Young's modulus treated by DOX in different concentrations

License and Terms: This is a supporting information file under the terms of the Creative Commons Attribution License (https://creativecommons.org/licenses/by/4.0). Please note that the reuse, redistribution and reproduction in particular requires that the author(s) and source are credited and that individual graphics may be subject to special legal provisions.
The license is subject to the *Beilstein Journal of Nanotechnology* terms and conditions: (https://www.beilstein-journals.org/bjnano/terms)

Table S1: Surface roughness of the four cell types.

Roughness	HDPE6-C7	AsPC-1	MIA-PaCa-2	BxPC-3
R_a (nm)	645.04	102.31	708.96	77.26

Table S2: The energy dissipation of different cell types.

Dissipation	HDPE6-C7	AsPC-1	BxPC-3	MIA-PaCa-2
W (nJ)	208.06	818.69	540.26	772.86

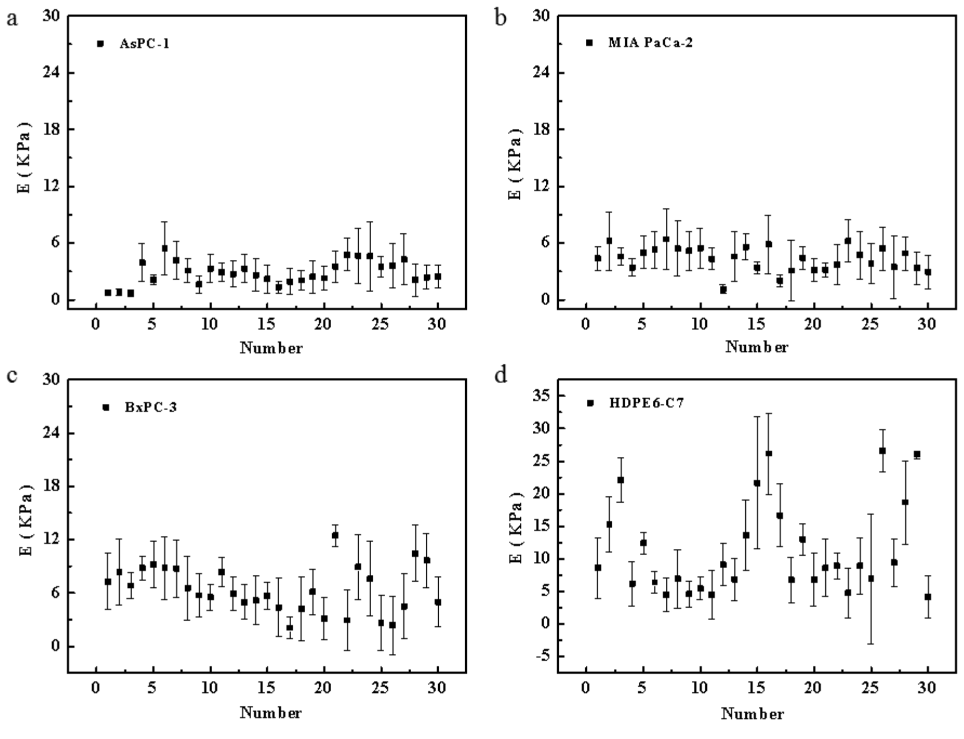

Figure S1: The point distribution of Young's modulus of (a) AsPC-1, (b) MIA-PaCa-2, (c) BxPC-3, and (d) HDPE6-C7.

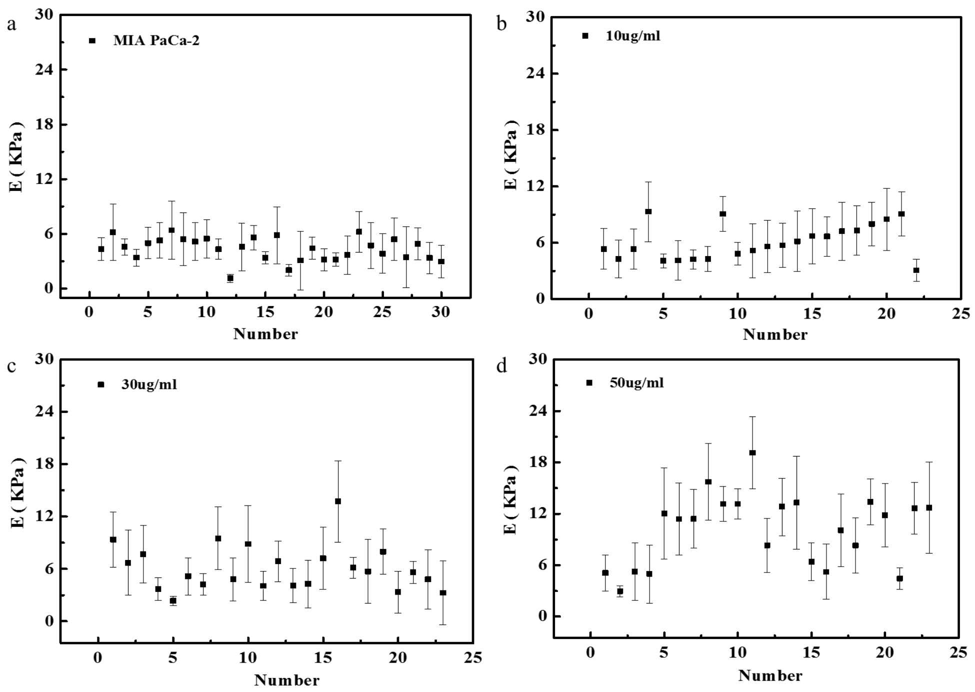

Figure S2: Point distribution of the Young's modulus of MIA PaCa-2 cells after treatment with DOX in different concentrations.
